# Changing Perceptions of End-of-Life Care Among Older Chinese Americans: Role of Acculturation

**DOI:** 10.1093/geroni/igaf122.3730

**Published:** 2025-12-31

**Authors:** Peiyuan Zhang, Jing Wang, Fei Sun, Xiaoyouxiang Li, Yaofeng Chen, Kaipeng Wang

**Affiliations:** University of Alabama, Tuscaloosa, Alabama, United States; University of New Hampshire, Durham, New Hampshire, United States; Michigan State University, East Lansing, Michigan, United States; University of Denver, Denver, Colorado, United States; huazhong university of science and technology, huazhong university of science and technology, Hubei, China (People’s Republic); University of Denver, Denver, Colorado, United States

## Abstract

Chinese Americans are one of the largest and fastest-growing immigrant groups in the United States, yet end-of-life (EOL) care remains disproportionately underutilized among this population. This study explores perceptions of end-of-life care and how they were shaped by acculturation among older Chinese Americans. Researchers conducted seven focus groups in person or via Zoom with 46 Chinese Americans aged 60 or above living in the United States in 2024. The study included three Mandarin (n = 22; mean years lived in the US: 18), two Cantonese (n = 9; mean years lived in the US: 33), and two English (n = 15; mean years lived in the US: 53) focus groups. All interviews were recorded, transcribed, and thematically analyzed using open coding. Major findings were (1) limited awareness of EOL care before immigrating to the U.S., (2) increased understanding of palliative and hospice care through the experiences of informal social networks post-immigration, (3) adaptation of traditional Chinese cultural views, such as filial piety, which influenced EOL preferences for life-prolonging care, (4) a shift toward greater autonomy and control in EOL decision-making, and (5) growing openness to discuss death and dying. Findings highlight the role of immigration history and acculturation in shaping Chinese American older adults’ evolving perceptions of end-of-life care, underscoring the need for clinicians to adopt culturally attuned communication strategies and decision-making support tools tailored to the values and experiences of immigrant older adults.

